# Identifying outstanding units in centralized systems: A DEA approach

**DOI:** 10.1371/journal.pone.0344454

**Published:** 2026-05-14

**Authors:** Zahra Cheraghali, Mohsen Rostamy-Malkhalifeh

**Affiliations:** 1 Department of Applied Mathematics, Faculty of Mathematical Sciences, Shahid Beheshti University, Tehran, Iran; 2 Faculty of Science, Science and Research Branch, Islamic Azad University, Tehran, Iran; Universidade de Lisboa Instituto Superior de Ciencias Sociais e Politicas, PORTUGAL

## Abstract

In centralized organizations, senior managers typically supervise multiple comparable decision-making units (DMUs), and improving overall efficiency depends on identifying units that performance has more impact on systems. Although Data Envelopment Analysis (DEA) has been extensively applied for performance evaluation in such environments, most traditional DEA-based approaches, such as super-efficiency, focus on local efficiency instead of the collective contribution of multiple DMUs. More recent centralized DEA formulations used to identify outstanding units partially remove this limitation; however, many of these models rely on predefined constants, which may lead to infeasible or unbounded optimization problems and reduce the robustness and interpretability of the results. This study proposes a new DEA-based mixed-integer nonlinear programming model to identify a subset of k outstanding DMUs from n units operating within a centralized system. The proposed model eliminates the need for any predefined constants and guarantees feasibility and boundedness. The proposed model is validated using two empirical. From a managerial perspective, the proposed approach provides a practical decision-support tool for centralized organizations.

## 1. Introduction

Centralized organizational structures are widely observed in both public and private sectors, have multi-branch firms operating across different regions or countries. In such systems, a senior manager typically supervises a large number of comparable decision-making units (DMUs). A key managerial challenge in these organizations is motivating local managers to improve not only their individual performance but also their collective contribution to organizational objectives. Because local decisions jointly determine system-wide outcomes, performance evaluation and incentive design in centralized systems must explicitly account for collective effects rather than focusing solely on individual units.

Data Envelopment Analysis (DEA) has been extensively used as a nonparametric approach for performance evaluation in multi-input–multi-output environments. Classical DEA models, such as the CCR and BCC, primarily focus on measuring the relative efficiency of individual DMUs. While these models are well suited for benchmarking and local performance assessment, they offer limited guidance for centralized decision-making, where the primary concern is improving overall system efficiency rather than optimizing the performance of individual units. To overcome this limitation, several extensions of DEA have been proposed, including super-efficiency models, which allow efficient DMUs to be ranked by excluding the evaluated unit from the reference set [1]. Despite their usefulness, super-efficiency models remain fundamentally local in nature and do not capture the joint impact of multiple DMUs on system performance.

Recognizing the need for system evaluation, a growing stream of literature has focused on centralized DEA models. Early contributions by Lozano and Villa [2] and Asmild et al. [3] proposed centralized DEA models that aggregate inputs and outputs across DMUs to evaluate overall efficiency and support resource allocation decisions. Varmaz et al. [4] further explored centralized planning and yardstick competition in multi-unit organizations. Although these approaches provide valuable insights into aggregate efficiency, they typically treat all DMUs symmetrically and do not explicitly identify those units whose performance disproportionately influences overall system outcomes. Consequently, their applicability for incentive design and targeted managerial interventions remains limited.

To solve this problem, Afsharian et al. [5] and Afsharian and Bogetoft [6] introduced DEA-based incentive systems for centrally managed organizations. In particular, Afsharian and Bogetoft [6] proposed a model that identifies a subset of outstanding DMUs based on their collective contribution to total organizational efficiency. This framework represents an important advancement by explicitly linking efficiency measurement with managerial motivation and incentive design in centralized systems. By focusing on the joint impact of selected DMUs, the model moves beyond purely local efficiency assessments and provides a more system-oriented perspective on performance evaluation.

Despite its conceptual appeal, the model proposed by Afsharian and Bogetoft [6] relies on predefined constants. As demonstrated in this study, the inclusion of such externally specified parameters may lead to infeasible or unbounded problems. Moreover, the choice of these constants is inherently arbitrary and may significantly affect the resulting efficiency scores and the identification of outstanding units. These issues reduce the robustness, interpretability, and practical applicability of the model, particularly in real-world managerial settings where parameter tuning is undesirable.

Motivated by these limitations, this paper proposes a new DEA-based methodology for identifying a subset of k outstanding DMUs from among n existing units within a centralized system. The proposed model eliminates the need for any predefined constants and guarantees both feasibility and boundedness. Methodologically, the model introduces a binary selection mechanism that directly links efficiency constraints to the selection of DMUs, while restricting the objective function to the outputs of the selected units.

By capturing collective performance effects in a robust and theoretically sound manner, the proposed framework provides a more comprehensive assessment of outstanding performance in centralized organizations. From a managerial perspective, the model offers a practical decision-support tool that enables central authorities to identify key performance-driving units, design incentive schemes aligned with system-level objectives, prioritize monitoring and benchmarking efforts, and guide resource allocation decisions.

The effectiveness of the proposed methodology is illustrated through two empirical case studies, and the results are compared with those obtained from existing DEA-based approaches. The findings demonstrate that the proposed model not only avoids infeasibility and unboundedness issues observed in earlier formulations, but also provides clearer and more interpretable insights into how exceptional DMUs contribute to overall organizational efficiency.

The remainder of this paper is organized as follows. Section 2 reviews related literature on DEA models and incentive mechanisms in centralized systems. Section 3 presents the proposed model and discusses its theoretical properties. In Section 4, we apply the model to two datasets and analyze the results and discuss managerial implications, and Section 5 concludes the paper with directions for future research.

## 2. Preliminaries and fundamentals

Let us consider a set of DMUs, denoted by N={1, 2,…,n}, each utilizing M={1, 2, …, m} inputs to produce S={1, 2, …, s} outputs. For each DMU_j_, Let $X_{j}=(x_{1j},\;x_{2j},\;\ldots ,\;x_{mj})\in {ℛ}^{m}$ and $Y_{j}=(y_{1j},\;y_{2j},\;\ldots ,\;y_{sj})\in {ℛ}^{s}$ be non-negative and non-zero vectors representing the inputs and outputs of *DMU*_*j*_, j∈N. The efficiency of a particular unit *DMU*_*o*_, denoted by *E*_*o*_, is evaluated by the classical input-oriented DEA model introduced by Charnes et al. [7]:


$$\begin{array}{c} E_{o}=\max\sum_{r\epsilon S}u_{r}y_{ro} \end{array}$$

$$\begin{array}{c} S.t.\;\sum_{i\epsilon M}v_{i}x_{io}=1 \end{array}$$

$$\begin{array}{c} \sum_{r\epsilon S}u_{r}y_{rj}-\sum_{i\epsilon M}v_{i}x_{ij}\leq 0,\;j\in N \end{array}$$

$$\begin{array}{c} u_{r},\;v_{i}\geq 0 \end{array}$$ (1)

In model (1), *v*_*i*_ and *u*_*r*_ are the weights of the i^th^ input and r^th^ output, respectively (for more details, see [8–11].

One of the methods to rank the efficient DMUs and find one outstanding DMU in DEA is the super-efficiency method [12], originally introduced by Andersen and Petersen [1]. In this method, the DMU under evaluation is excluded from the reference set that defines the efficient frontier. By considering the super-efficiency condition, model (1) is then reformulated as model (2):


$$\begin{array}{c} E_{o}^{AP}=\max\sum_{r\epsilon S}u_{r}y_{ro} \end{array}$$

$$\begin{array}{c} S.t.\;\sum_{i\epsilon M}v_{i}x_{io}=1 \end{array}$$

$$\begin{array}{c} \sum_{r\epsilon S}u_{r}y_{rj}-\sum_{i\epsilon M}v_{i}x_{ij}\leq 0,\;j\in N,\;j\neq o \end{array}$$

$$\begin{array}{c} u_{r},\;v_{i}\geq 0 \end{array}$$ (2)

The super-efficiency model allows efficient DMUs to be ranked beyond the frontier for further reading, see Zanboori et al. [13]. However, this approach primarily captures local efficiency rather than the global or collective effect of DMUs on overall system performance. When identifying multiple outstanding units, this method must be executed iteratively — removing each identified DMU from the dataset before re-running the model. Such a stepwise process does not reflect the combined influence of selected DMUs on the organization as a whole. To evaluate the overall efficiency of a system comprising multiple units under centralized control, the centralized DEA model is often employed. Its general form can be expressed as follows:


$$\begin{array}{c} E_{overall}=\max\sum_{j\in N}\sum_{r\epsilon S}u_{r}y_{rj} \end{array}$$

$$\begin{array}{c} S.t.\;\sum_{j\in N}\sum_{i\epsilon M}v_{i}x_{ij}=1 \end{array}$$

$$\begin{array}{c} \sum_{r\epsilon S}u_{r}y_{rj}-\sum_{i\epsilon M}v_{i}x_{ij}\leq 0,\;j\in N \end{array}$$

$$\begin{array}{c} u_{r},\;v_{i}\geq 0 \end{array}$$ (3)

In model (3), the objective function and the first constraint consider the aggregate levels of each output and input, while the remaining constraints are similar to those in model (1). Since model (3) is input-oriented, it aims to minimize total inputs while maintaining output levels. Several centralized DEA formulations have been proposed in the literature [e.g., 4,3,Lozana and Villa, 2004,^5^ can be referred to].

To extend the concept of super-efficiency to centralized systems, model (3) can be modified as follows:


$$\begin{array}{c} E_{overall}^{AP}=\max\sum_{j\in N}\sum_{r\epsilon S}u_{r}y_{rj} \end{array}$$

$$\begin{array}{c} S.t.\;\sum_{j\in N}\sum_{i\epsilon M}v_{i}x_{ij}=1 \end{array}$$

$$\begin{array}{c} \sum_{r\epsilon S}u_{r}y_{rj}-\sum_{i\epsilon M}v_{i}x_{ij}\leq 0,\;j\in N,\;j\neq o \end{array}$$

$$\begin{array}{c} u_{r},\;v_{i}\geq 0 \end{array}$$ (4)

Although this extension provides a means to identify efficient units within a centralized setting, it neglects the collective impact of multiple DMUs. To address this limitation, Afsharian and Bogetoft [6] proposed a model that identifies a subset of k outstanding DMUs (*k* ≤ *n*) that collectively contribute most to the overall efficiency of the system. Their model is formulated as:


$$\begin{array}{c} E_{overall}^{k}=\max\sum_{j\in N}\sum_{r\epsilon S}u_{r}y_{rj} \end{array}$$

$$\begin{array}{c} S.t.\;\sum_{j\in N}\sum_{i\epsilon M}v_{i}x_{ij}=1 \end{array}$$

$$\begin{array}{c} \sum_{r\epsilon S}u_{r}y_{rj}-\sum_{i\epsilon M}v_{i}x_{ij}\leq \left(1-\tau_{j}\right)\omega \;,\;j\in N \end{array}$$

$$\begin{array}{c} \sum_{j\in N}\tau_{j}=n-k \end{array}$$

$$\begin{array}{c} u_{r},\;v_{i}\geq 0\;\forall i,r \end{array}$$ (5)

[For more details, see 14–16]. While this model effectively incorporates the selection of multiple outstanding DMUs, it may still yield unbounded or infeasible results under certain data configurations. To overcome these drawbacks, we develop an enhanced model in the next section that ensures both feasibility and boundedness while retaining the ability to evaluate the joint impact of selected DMUs.

## 3. Proposed model

To identify the subset of k DMUs that exert the greatest influence on the overall efficiency of the centralized system, we introduce a binary selection variable $\tau_{j}$, defined as follows:


$$\begin{array}{c} \tau_{j}=\left\{ \begin{array}{c} 1\;if\;{DMU}_{j}\;is\;selected\;\\ 0\;Otherwise\; \end{array}\right. \end{array}$$ (6)

Using this binary variable, we propose the following mixed-integer nonlinear programming (MINLP) model to identify the *k* outstanding DMUs that contribute most significantly to total system efficiency:


$$\begin{array}{c} {\hat{E}}_{overall}^{k}=\max\sum_{j\in N}\sum_{r\epsilon S}u_{r}y_{rj}\tau_{j} \end{array}$$

$$\begin{array}{c} S.t.\;\sum_{j\in N}\sum_{i\epsilon M}v_{i}x_{ij}=1 \end{array}$$

$$\begin{array}{c} \sum_{r\epsilon S}u_{r}y_{rj}-\sum_{i\epsilon M}v_{i}x_{ij}\leq \tau_{j}\;,\;j\in N \end{array}$$

$$\begin{array}{c} \sum_{j\in N}\tau_{j}=k \end{array}$$

$$\begin{array}{c} \tau_{j}\in \left\{0,1\right\}\;\forall j \end{array}$$

$$\begin{array}{c} u_{r},\;v_{i}\geq 0\;\forall i,r \end{array}$$ (7)

Model (7) improves upon the model of Afsharian and Bogetoft [6] by eliminating the need for any predefined constants. In the earlier formulation, the constant ω must be selected exogenously, and inappropriate values may lead to infeasible or unbounded optimization problems. In contrast, the proposed model derives the selection of outstanding DMUs endogenously through the optimization structure itself, thereby enhancing both robustness and interpretability.

From a methodological perspective, Model (7) reformulates the selection mechanism by directly linking the efficiency constraints to the binary selection variable $\tau_{j}$. When $\tau_{j}=0$, the corresponding constraint forces the weighted outputs of *DMU*_*j*_ to be dominated by its weighted inputs, ensuring that non-selected DMUs do not contribute positively to the objective function.

When $\tau_{j}=1$, the constraint reduces to the standard DEA efficiency condition, allowing the DMU to contribute to overall efficiency. Importantly, all DMUs remain part of the reference technology through the constraint set; non-selected units are not removed from the production possibility set, which addresses potential ambiguity associated with the term “elimination”.

A key property of the proposed model is that feasibility and boundedness are guaranteed for any k∈{0,1,…,n}. Feasibility follows from the existence of non-negative weights satisfying the normalization constraint, while boundedness is ensured by the restriction of the objective function to the outputs of selected DMUs combined with the fixed normalization of aggregate inputs. As a result, the objective value is inherently bounded above, and the model avoids the infeasibility and unboundedness issues observed in previous formulations.

Conceptually, Model (7) differs from Model (5) in how overall efficiency is evaluated. While Model (5) includes all DMUs in the computation of total efficiency—even those not identified as outstanding—the proposed model restricts the objective function exclusively to the performance of the selected DMUs ($\tau_{j}=1$). Consequently, the resulting efficiency measure explicitly reflects the joint contribution of the chosen subset of DMUs to system-level performance, rather than diluting this effect by aggregating over the entire set of units.

By directly capturing the aggregated impact of the most influential DMUs, the objective function in Model (7) provides a more accurate and interpretable measure of collective performance in centralized systems. This model enables decision-makers to assess how different combinations of outstanding units influence overall efficiency and supports more informed managerial decisions regarding incentive design and resource allocation.

Now, we formally discuss the feasibility and boundedness of the proposed model (7) for any admissible value of k.

- **Feasibility.**

For any k∈{0,1,…,n}, model (7) is feasible. To see this, observe that the normalization constraint $\;\sum_{j\in N}\sum_{i\epsilon M}v_{i}x_{ij}=1$, admits at least one feasible solution for non-negative weights *v*_*i*_, provided that the aggregate input vector is strictly positive, which is a standard assumption in DEA. Given such weights, setting *u*_*r*_ = 0 for all r∈S and selecting any subset of k DMUs by assigning $\tau_{j}=1$ for the selected units and $\tau_{j}=0$ otherwise satisfies all remaining constraints. Hence, the feasible region of model (7) is non-empty for all admissible values of k.

- **Boundedness.**

The objective function of model (7) is bounded above. This follows from two key structural properties. First, the normalization constraint fixes the weighted sum of aggregate inputs to unity, which restricts the scale of the input weights. Second, for each *DMU*_*j*_, the constraint $\sum_{r\epsilon S}u_{r}y_{rj}-\sum_{i\epsilon M}v_{i}x_{ij}\leq \tau_{j}$ implies that the weighted outputs of any selected DMU ($\tau_{j}=1$) are bounded above by $1+\sum_{i\in M}v_{i}x_{ij}$. Since the number of selected DMUs is fixed at k and all variables are non-negative, the objective function cannot grow unboundedly.

Taken together, these properties ensure that model (7) is both feasible and bounded. This analytical argument complements the numerical evidence presented in the subsequent section and distinguishes the proposed model from earlier models that rely on predefined constants and may suffer from infeasibility or unboundedness.

In the following section, the limitations of Model (5) are illustrated through a numerical example. Subsequently, the proposed model is applied to the dataset used by Varmaz et al. [4] to validate its effectiveness, followed by a comparative analysis of the results.

## 4. Case study

To compare the proposed model with existing DEA-based approaches, we apply it to two datasets. The first dataset is a small illustrative example, while the second dataset is taken from Varmaz et al. [4], which has been widely used in the centralized DEA literature.

- **Example 1:**

The first dataset, reported in Table 1, consists of six DMUs, each using two inputs to produce two outputs. The objective is to identify subsets of outstanding DMUs for different values of k. Specifically, we consider k = 0,1,2,3 and compare the results obtained from the model of Afsharian and Bogetoft [6] (Model (5)) and the proposed model (Model (7)). For Model (5), the predefined constant is set to $\omega =10000000000000=10^{13}$, a value sufficiently large to satisfy the intended logic of the model, as commonly assumed in practice.

**Table 1:** Data 1.

DMU (Unit)	*x*_1_	*x*_2_	*y*_1_	*y*_2_
1	10000	30000	1	1
2	10000	15000	1	2
3	15000	10000	2	3
4	15000	30000	2	1
5	20000	20000	3	2
6	10000	10000	2	1

The results are summarized in Table 2. When *k* = 0, no DMU is selected as outstanding. In this case, the objective value of the proposed model is equal to zero, reflecting the fact that no unit contributes to the objective function. This result should be interpreted as a benchmark scenario rather than as an indication that efficiency disappears from the system. By contrast, Model (5) still yields a positive efficiency score because it continues to aggregate performance across all DMUs.

**Table 2:** Overall error values for different *k.*

*k*	$E_{overall}^{k}\;$	${\hat{E}}_{overall}^{k}\;$
0	0.83	0
1	0.88 {3}	0.20 {5}
2	2.59 {2,5}	0.63 {2,3}
3	Infeasible or unbounded	0.88 {2,3,5}

For *k* = 1 and *k* = 2, both models are able to identify subsets of outstanding DMUs, although the selected units and efficiency values differ. These differences highlight the conceptual distinction between the two formulations: Model (5) evaluates overall efficiency using all DMUs, whereas the proposed model restricts the objective function to the selected units only, thereby isolating their collective contribution.

A key observation arises for *k* = 3. As shown in Table 2, Model (5) becomes infeasible or unbounded, despite the large value assigned to ω. This behavior illustrates the sensitivity of the model to the predefined constant and confirms the concerns raised in the literature. In contrast, the proposed model remains feasible and bounded and successfully identifies three outstanding DMUs. This result empirically supports the theoretical properties discussed in the previous section.

- **Example 2:**

The second dataset, presented in Table 3, consists of 16 DMUs and four variables (three inputs and one output), originally studied by Varmaz et al. [4]. We apply the super-efficiency model, the centralized super-efficiency model, the model of Afsharian and Bogetoft [6], and the proposed model to identify outstanding DMUs for k=1,2,…,6. The corresponding results are reported in Table 4.

**Table 3:** Data 2.

DMU (Unit)	PEX	IEX	IIN	OIN
1	1532.00	2769.00	110920.00	123100.00
2	998.00	1757.00	552900.00	77800.00
3	853.00	1220.00	238400.00	46400.00
4	180.00	378.00	632000.00	1330000.00
5	584.00	876.00	184700.00	29700.00
6	498.00	2080.00	268900.00	52400.00
7	261.00	395.00	135800.00	20300.00
8	609.00	883.00	268800.00	35200.00
9	222.00	528.00	791000.00	14900.00
10	264.00	700.00	856000.00	1930000.00
11	1078.00	1448.00	187300.00	61100.00
12	222.00	503.00	770000.00	21700.00
13	258.00	412.00	520000.00	13800.00
14	696.00	1099.00	283600.00	44300.00
15	176.00	361.00	477000.00	10400.00
16	236.00	301.00	724000.00	15900.00

**Table 4:** Result of Data 2.

*k*	*E* ^*AP*^	$E_{overall}^{AP}\;$	$E_{overall}^{k}\;$	${\hat{E}}_{overall}^{k}\;$
1	{1}	0.856 {12}	0.856 {12}	0.23 {1}
2	{1,6}	0.853 {7,12}	0.861 {6,12}	0.37 {1,2}
3	{1,6,12}	0.868 {1,7,12}	0.997 {1,2,7}	0.46 {1,2,7}
4	{1,6,7,12}	0.925 {1,2,7,12}	1.023 {1,2,6,7}	0.57 {1,2,7,8}
5	{1,2,6,7,12}	0.923 {1,2,7,12,16}	1.222 {1,2,7,8,14}	0.77 {1,2,7,8,14}
6	{1,2,6,7,12,16}	0.929 {1,2,7,12,14,16}	1.274 {1,2,6,7,8,14}	0.84 {1,2,7,8,11,14}

Several important observations can be drawn from these results. First, when k = 1, the proposed model and the classical super-efficiency approach identify the same outstanding unit. This outcome is expected, as selecting a single outstanding DMU reduces the problem to a local efficiency comparison. However, as *k* increases, the advantages of the proposed model become more apparent.

Specifically, the efficiency values obtained from the proposed model increase monotonically with k, reflecting the growing collective contribution of the selected units. In contrast, the efficiency scores produced by Model (5) exceed unity and exhibit greater variability, which complicates interpretation from a managerial perspective. This difference stems from the fact that Model (5) aggregates performance across all DMUs, while the proposed model isolates the contribution of the selected subset.

Moreover, the proposed model remains feasible and numerically stable for all values of k considered. In several cases (e.g., k = 3 and k = 5), the proposed model identifies the same outstanding DMUs as Model (5), suggesting consistency in identifying key performance-driving units. However, unlike Model (5), the proposed model avoids infeasibility and provides bounded, interpretable efficiency scores for all scenarios.

From a managerial perspective, these results offer clear insights. By varying k, decision-makers can examine how the size and composition of the selected subset influence overall system efficiency. This flexibility enables managers to design incentive schemes, prioritize monitoring efforts, and allocate resources based on the collective impact of outstanding units rather than relying on isolated efficiency rankings.

Overall, the numerical examples demonstrate that the proposed model provides a more stable, robust, and interpretable framework for identifying groups of DMUs with a substantial influence on system-level efficiency. By eliminating predefined constants and explicitly capturing collective effects, the model addresses key limitations of existing DEA-based approaches and offers a practical decision-support tool for centralized organizations.

All models were implemented using GAMS. The proposed model is a mixed-integer nonlinear programming (MINLP) problem due to the presence of binary selection variables and bilinear terms involving weights and selection indicators.

For the datasets considered in this study, the proposed model converged reliably to optimal solutions for all tested values of k. The average computation time was approximately 40 seconds for the both datasets. Although the computational burden increases with the number of DMUs due to the combinatorial nature of the selection problem, the results indicate that the proposed model remains tractable for moderate-sized datasets. This suggests that the model is suitable for practical applications in centralized organizational settings, where the number of DMUs is typically limited.

## 5. Conclusion

This study developed a novel DEA-based framework for identifying outstanding decision-making units in centralized organizational systems. The proposed model overcomes a fundamental limitation of existing approaches by eliminating the need for predefined constants, which are known to induce infeasibility or unboundedness under certain data configurations. In contrast, the new model guarantees feasibility and boundedness for all admissible values of k, as established through analytical arguments and confirmed by numerical experiments.

Beyond its theoretical soundness, the proposed model introduces a conceptually important shift in the evaluation of performance in centralized systems. Rather than aggregating efficiency across all units, the model explicitly isolates and quantifies the collective contribution of a selected subset of DMUs to overall system efficiency. This distinction enables a more transparent and interpretable assessment of group-level performance.

The empirical analyses demonstrate that the proposed approach yields stable and consistent efficiency measures across different values of k, even in scenarios where existing models fail or produce implausible results. The ability of the model to remain well-behaved under varying data configurations highlights its robustness and practical relevance.

From a managerial standpoint, the framework offers actionable insights for senior decision-makers operating in centralized environments. By identifying combinations of units that exert the greatest influence on total efficiency, the model supports the design of incentive schemes, prioritization of managerial attention, and more effective allocation of limited resources. Importantly, the framework allows managers to explore trade-offs between the number of selected units and their collective impact on organizational performance.

Several promising directions for future research emerge from this work. Extensions to stochastic or fuzzy data environments would enhance applicability under uncertainty, while adaptations to hierarchical or networked organizational structures could broaden its relevance. Additionally, dynamic formulations that capture performance evolution over time represent a natural and valuable extension of the proposed approach.
